# Scapulothoracic Anatomy and Snapping Scapula Syndrome

**DOI:** 10.1155/2013/635628

**Published:** 2013-11-28

**Authors:** Rachel M. Frank, Jose Ramirez, Peter N. Chalmers, Frank M. McCormick, Anthony A. Romeo

**Affiliations:** Section of Shoulder and Elbow Surgery, Department of Orthopaedic Surgery, Rush University Medical Center, Chicago, IL 60612, USA

## Abstract

The scapulothoracic articulation is a sliding junction between the deep aspect of the scapula and thoracic rib cage at the levels of ribs 2 through 7. Motion at this articulation is dynamically stabilized by a variety of muscular attachments, allowing for controlled positioning of the glenoid to assist in glenohumeral joint function. A thorough understanding of the complex anatomic relationships, including the various muscles, and bursa, is critical to the evaluation of patients presenting with scapulothoracic disorders. The snapping scapula syndrome is caused by either osseous lesions or scapulothoracic bursitis and can be difficult to recognize and treat. The purpose of this review is to discuss the anatomy of the scapulothoracic articulation with an emphasis on the pathology associated with snapping scapula syndrome.

## 1. Introduction

The scapulothoracic articulation is a complex anatomical structure that plays a substantial role in overall shoulder function. The osseous, ligamentous, and muscular periscapular relationships are intricate. While scapulothoracic pathology is uncommon, a thorough appreciation of the anatomy, including the various muscular relationships and bursal planes, is critical to the evaluation of patients presenting with scapulothoracic disorders [1]. Snapping scapula syndrome is caused by either osseous lesions or scapulothoracic bursitis, and appropriate recognition and treatment of these disorders is dependent on a solid foundation in periscapular anatomy [2, 3]. The purpose of this review is to discuss the anatomy of the scapulothoracic articulation with an emphasis on the pathology associated with snapping scapula syndrome.

## 2. Anatomical Description of the Scapula

**Figure 1:**
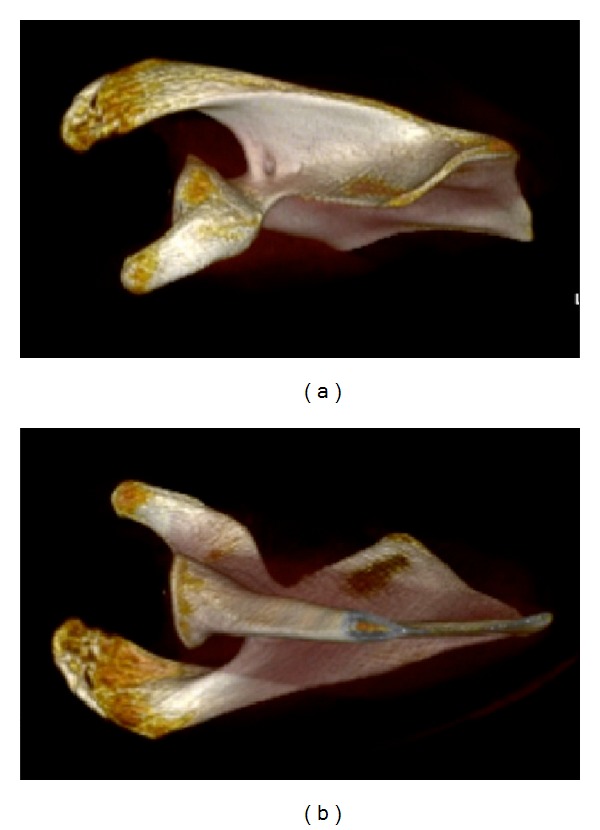
Three-dimensional reconstruction of the scapula demonstrating the (a) superior and (b) inferior osseous morphology of the scapula.

**Figure 2:**
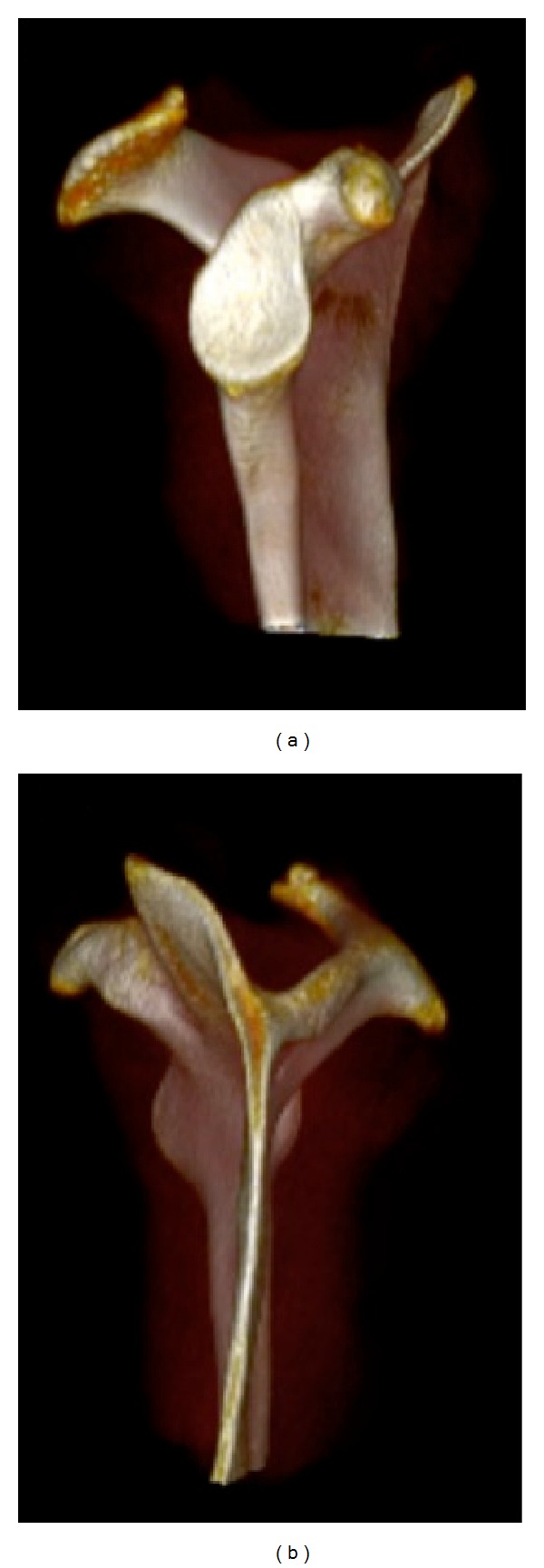
Three-dimensional reconstruction of the scapula demonstrating the (a) lateral (glenoid face) and (b) medial osseous morphology of the scapula.

The scapula is a flat bone (Figures 1 and 2) that rests on the posterolateral aspect of the thoracic cavity overlying ribs 2 through 7 [1, 3]. The scapula serves as a site for multiple muscular origins and insertions [1] and is thin and triangular shaped with three distinct borders (superior, axillary, and vertebral) and three angles (superomedial, inferomedial, and lateral (glenoid)) [4] (Figures 1 and 2). The superomedial angle, formed by the superior border and the vertebral border of the scapula, normally measures 124 to 162 degrees [5]. These anatomical variations in the superomedial angle may have clinical implications in the development of snapping scapula syndrome [5]. The anterior surface of the scapula is undulating. Scapular thickness ranges from 10.5 to 26.7 mm [5].

The most medial border of the scapula (vertebral border) is approximately 5 cm from the vertebral column, although this distance varies depending on scapular protraction and retraction (Figures 1 and 2). The long axis of the scapula lies in a plane that is 30 to 45 degrees anterior to the coronal plane because of the curve of the rib cage [6, 7]. The scapula has several important osseous features, including the acromion, coracoid process, spine [1], and glenoid fossa. The acromion serves as a lever arm for the deltoid [1] and articulates with the lateral end of the clavicle at the acromioclavicular joint. The lateral aspect of the acromion partly overlies the rotator cuff. Impingement of the rotator cuff on the acromion may cause degeneration of the rotator cuff [8]. The shape of the acromion has been graded as curved (concave), flat, hooked, and inverted (convex) [8, 9]. The down-curved acromion has been associated with impingement and rotator cuff pathology [9–11]. The scapular spine separates the supraspinatus fossa from the infraspinatus fossa and extends from superiorly and laterally from the medial edge of the scapula, serving as the attachment of the trapezius and posterior deltoid muscles. The coracoid process originates from the upper border of the scapula, medial to the glenoid, and is tilted anterolateral approximately 120 to 160 degrees [5]. Two coracoclavicular ligaments (conoid and the trapezoid) connect the superior surface of the coracoid process and the clavicle. The coracohumeral and coracoacromial ligaments also attach to the coracoid process. Additionally, the coracoid process serves as the attachment of the coracobrachialis muscle, short head of the biceps brachii muscle, and the pectoralis minor muscle.

**Figure 3:**
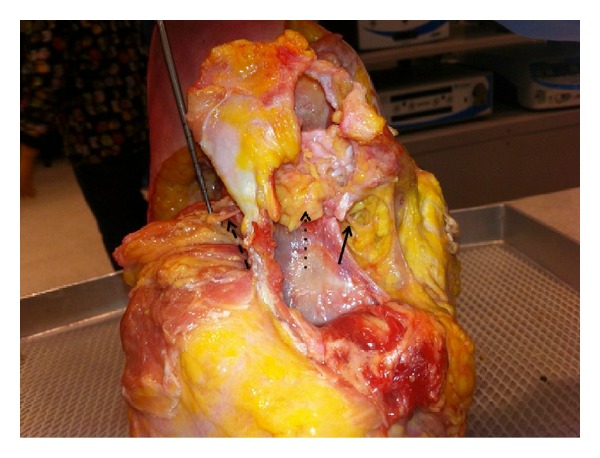
Photograph of the shoulder in the lateral decubitus position (viewed from superior with the anterior aspect of shoulder to the right) demonstrating dissection of the suprascapular nerve. The proximal portion of the nerve is marked with the solid arrow; the supraspinatus is flipped medially with the nerve running through adipose tissue marked with a dotted arrow between the acromion and the spine. The distal aspect of the nerve, marked with a dashed arrow, is seen on the left side of the specimen going into the infraspinatus.

The scapular notch is a useful anatomical landmark in identifying important neurovascular structures that are closely associated with the scapula. The notch is found at the base of the coracoid process along the scapular spine where it is spanned by the transverse scapular ligament. Notably, the suprascapular artery courses over the transverse scapular ligament to supply the infraspinatus and supraspinatus muscles, while the suprascapular nerve courses below the ligament to innervate the infraspinatus and supraspinatus [12, 13]. Compression of the suprascapular nerve (i.e., due to a ganglion cyst) can occur both proximally, at the level of the scapular notch, and distally, at the level of the spinoglenoid notch, which is an indentation in the scapula at the convergence of the acromion, lateral edge of the spine, and neck of the scapula. Compression in the suprascapular notch leads to weakness of both the supraspinatus and infraspinatus, while compression more distally in the spinoglenoid notch leads only to weakness of the infraspinatus, as the branch to the supraspinatus has already been given off (Figure 3).

The glenoid is a concave process on the lateral aspect of the scapula that articulates with the humeral head. As described above, the glenoid is separated from the acromion by the spinoglenoid notch. The articular surface of the glenoid is 1/3 to 1/4 the size of the humeral head [1], and its surface area and depth are increased by the circumferential fibrocartilaginous labrum. The glenoid is retroverted relative to the scapular plane by about 2 to 3 degrees [14, 15], although native anteversion has been described [16]. The face of the glenoid has 10 to 15 degrees of upward inclination tilt relative to the medial scapular border [6].

The scapula has a total of 17 muscular attachments (Table 1). The scapulohumeral muscles include the supraspinatus, infraspinatus, teres minor, teres major, deltoid, long and short heads of the biceps brachii, long head of the triceps brachii, and coracobrachialis. These muscles help to position the arm in space. As there are no osseous or ligamentous connections between the scapula and the thorax, the scapula is held in place and positioned by its muscular attachments to axial skeleton, which include the trapezius, serratus anterior, rhomboid major, rhomboid minor, levator scapulae, subclavius, and pectoralis minor muscles [17]. The deep fascia of trapezius and sternocleidomastoid also contribute to passive stability by connecting the head, clavicle, and scapular spine [6]. Of note, the muscular attachments to the clavicle also play a critical role in scapular function as the clavicle acts as a strut to hold the lateral aspect of the scapula lateral and posterior away from the body. Much of the periscapular musculature functions by pushing or pulling against the mobile fulcrum of the coracoclavicular and acromioclavicular articulations.

The trapezius muscle is a tent-like muscle [6] that provides passive scapular stabilization, active elevation of the lateral angle, scapular retraction, and upward scapular rotation [18]. The trapezius is composed of descending, ascending, and transverse fibers [6]. The descending fibers from the occipital protuberance and the nuchal ligament are directed towards the lateral clavicle, which serves more to elevate and rotate the scapula, while the horizontal fibers originate from the C7–T3 spinous processes and attach on the acromion and lateral scapular spine, serving more to retract the scapula [6]. Fibers ascending from the T3–T12 spinous processes also attach to the medial aspect of the scapular spine, serving as scapular retractors and depressors. Electromyographic evidence suggests that the trapezius may provide dynamic stability to the scapula while the arm is swinging, for example, during walking [19]. The rhomboid muscles retract and elevate the scapula. The minor and major muscles originate from the C6-C7 and T1–T4 spinous processes, respectively, and attach above and below the medial aspect of the scapular spine [6]. The levator scapulae contributes to elevation and rotation of the scapula. Its fibers are attached to the transverse processes of C1–C4 and insert on the superior angle of the spine [6].

The fibers of the serratus anterior originate anteriorly from ribs 1–9 and attach along the anterior aspect of the medial border of the scapula from the inferior to superior angle [6]. In addition to protraction and upward rotation, the serratus anterior holds the medial angle against the thoracic cage. Palsy of this muscle therefore leads to prominence, or “winging” of the medial angle. The serratus anterior and the pectoralis minor work together to protract the scapula.

The pectoralis minor originates from the anterior aspect of ribs 3–5 to attach to the medial aspect of the coracoid process, and participates in protraction, downward rotation, and depression of the scapula [6]. The subclavius originates from the medial aspect of the first rib and attaches to the inferior surface of the clavicle, pulling the clavicle medially towards the sternum, thereby stabilizing the sternoclavicular joint [6].

## 3. Movement of the Glenohumeral and Scapulothoracic Joints

The glenohumeral joint is the most mobile joint in the body, owing to its remarkable flexibility, to the lack of osseous constraint, and to the concerted actions of the 4 shoulder articulations, including the sternoclavicular, acromioclavicular, glenohumeral, and scapulothoracic joints [17]. In conjunction with multiple associatedmuscular and ligamentous components, the shoulder complex allows for coordinated movement of the clavicle, humerus, and scapula [6, 7].

The scapulothoracic “articulation” differs from the three other joints of the shoulder complex, as there is no articular cartilage, synovium, or capsule, but is a series of bursal and muscular planes, which allow sliding [2]. Beyond its attachments via the acromioclavicular and sternoclavicular joints, the scapula does not have any other attachments to the thorax [7]. Instead, the scapulothoracic joint is defined by soft tissue apposition, namely, that of the subscapularis muscle which spreads across the concave ventral scapula, lying over the serratus anterior muscle [7, 17]. The serratus anterior attaches over the 2nd through 7th ribs in the anterolateral and posterior thoracic cage. During motion of the shoulder, the subscapularis muscle glides over the underlying layers of the serratus anterior [7, 17]. During normal shoulder motion, the scapula translates by the varying combinations of forces exerted on the scapula by its muscular attachments that produce protraction, retraction, elevation, depression, and rotation [7, 17]. Protraction occurs with forward movement of the scapula away from the midline vertebral column, combined with internal rotation and anterior tilting [6]. Retraction is the opposite of these motions.

Movement of the glenohumeral joint is controlled by the multiaxial articulation of the concave humeral head with the concave glenoid fossa of the scapula [7]. As it articulates with the glenoid, the humerus rolls, spins, and slides [7] in order to adduct, abduct, extend, flex, and rotate the humerus [7, 17]. Changes in the position of the scapula change the relative position of the glenoid fossa and influence glenohumeral joint articulation.

Obligate external rotation of the humerus with abduction prevents impingement of the greater tuberosity on the coracoacromial arch [20]. Internal rotation of the shoulder is mostly a product of motion of the glenohumeral joint, with minimal contribution from the scapulothoracic articulation. Most people with normal shoulder function will use about 15 degrees of scapulothoracic internal rotation to care for themselves; in the setting of a glenohumeral fusion this increases to 51 degrees of scapulothoracic internal rotation [6].

Full abduction is accomplished via the coordinated movement of several joints. Through the first 30 degrees of abduction, the position of the scapula is relatively unchanged [7] as much of the motion takes places at the glenohumeral joint. With continued abduction, the scapula and clavicle rotate counter-clockwise about an axis that extends from the sternoclavicular joint to the medial edge of the scapular spine, contributing approximately 40 degrees to abduction [18]. At approximately 100 degrees of abduction, the sternoclavicular joint becomes rigid and continued lateral (counter-clockwise) rotation of the scapula occurs about the acromioclavicular joint, contributing an additional 20 degrees to abduction [18, 21]. As full abduction is reached, the trapezoid ligament will also become rigid, stopping rotation about the acromioclavicular joint [7, 21].

## 4. Normal Scapulothoracic Articulation

The dynamic interactions at the scapulothoracic joint are facilitated by intervening layers of multiple muscles and bursae. Williams et al. [22] described three levels/layers incorporating the muscle and bursal tissue, including superficial, intermediate, and deep. The superficial layer includes the trapezius and latissimus dorsi muscle. The intermediate layer is formed by the rhomboid major and minor and levator scapulae muscles. A number of important neurovascular structures are also found within the intermediate layer. The spinal accessory nerve, which innervates the trapezius, can be found lateral to the levator scapulae muscle adjacent to the superomedial angle [22]. The dorsal scapular nerve, which innervates the levator scapulae and rhomboid muscles, can be found under the levator scapulae muscle traveling parallel to the medial scapular border [23]. The suprascapular nerve can also be found in the intermediate layer as it courses across the superomedial angle in the direction of the suprascapular notch [24]. The transverse cervical artery branches at the level of the levator scapulae muscle [25]. The deep layer is formed by subscapularis and serratus anterior muscles.

Kuhn et al. [26] reported the locations of six bursae (2 major, 4 minor) participating in scapulothoracic articulation. Two of these bursae, including the scapulothoracic (infraserratus) bursa and subscapularis (supraserratus) bursa are the primary physiologic bursae [22, 27, 28], and are found in the deep layer. The subscapularis bursa, found between the subscapularis and serratus anterior muscles, is, on average, 5.3 × 5.3 cm when present [22]. The infraserratus bursa is found under the serratus anterior, overlying the posterolateral chest wall and is on average 9.0 × 7.4 cm [22].

The 4 minor bursae are not consistently found and are often a result of abnormal scapulothoracic articulation [27]. These are typically found along the inferior angle of the spine, at the superomedial border of the scapula, either above or below the serratus anterior, or deep to the trapezius muscle at the medial base of the scapular spine [29]. The bursa of the superomedial border and inferior angle are frequently pathologic and responsible for symptom generation [25, 30].

One superficial bursa has also been described to be located between the latissimus dorsi and the inferomedial angle of the scapula approximately 1.9 × 2.4 cm in size [22]. One intermediate level bursa, called the scaphotrapezial (trapezoid) bursa, has been described to be located between the superomedial scapula and the trapezius muscles in the intermediate level, approximately 4.3 × 2.7 cm in size.

## 5. Abnormal Scapulothoracic Articulation

Abnormal motion of the scapula on the underlying thorax is the basis for the development of the snapping scapula syndrome [2, 3]. Patients with scapulothoracic bursitis often present without a history of trauma or injury to the shoulder [31], although they may report a history of repetitive overhead activity such as swimming or pitching, gymnastics, or weightlifting [32]. Patients may also report neck pain or debilitating shoulder pain with possible numbness or tingling in the extremity [31], whereas a subset of patients will report a painless sensation of snapping with shoulder motion, other patients will report activity-related pain directly associated with snapping or crepitus [27, 33]. Physical examination may reveal a tender, fluctuant mass around the medial border of the scapula [34], scapulothoracic crepitation with active or passive movement of the shoulder [31], as well as possible atrophy and/or weakness of periscapular musculature.

Bursitis and crepitus at the scapulothoracic articulation typically results from either repetitive motion of the scapulothoracic joint in the appropriate anatomic and physiologic milieu [35–37] from a variety of soft tissue and/or bony abnormalities of the medial aspect of the scapula [38]. One review of 89 cases of scapulothoracic bursitis found that an anatomic abnormality was responsible for symptoms in 43% of the cases [30]. The Luschka Tubercle and excessive anterior angulation of the superior angle of the scapula are examples of skeletal abnormalities. The Luschka Tubercle is a hook-shaped extension of the superomedial border of the scapula that may cause irregular scapulothoracic articulation [39]. Changes in the thorax due to kyphosis can also alter the scapulothoracic articulation [26]. The presence of an osteochondroma, a benign cartilage tumor [40], can also cause scapulothoracic crepitus [31]. An osteochondroma is the most common benign tumor of the scapula [41], with a reported incidence of 3 to 4.6% [42–44]. These lesions usually arise over the ventral surface of the bone. One study implicated osteochondroma in 16% of reviewed cases of snapping scapula syndrome [31]. Chondrosarcoma can also arise in the scapula, and can affect scapulothoracic articulation in rare cases [45]. Calcific spurring of the superomedial angle of the scapula from chronic trauma or avulsion of the levator scapulae muscle has also been implicated in cases of scapulothoracic bursitis [46].

While rare, abnormal scapula motion leading to scapulothoracic bursitis can also result from nerve injury, muscle overuse, and muscle imbalance leading to impaired control of scapular motion [46, 47]. Impaired scapular motion, otherwise known as scapular dyskinesis [48], has been identified in patients with glenohumeral joint pathologies, including shoulder instability [49] and rotator cuff pathology [50, 51]. Muscular atrophy of the serratus anterior and subscapularis, as a result of long thoracic nerve palsy and glenohumeral fusion, respectively, has been reported as causes of scapulothoracic dyskinesis [52]. In a series of 100 patients treated with resection of the 1st rib for thoracic outlet syndrome, 15 went on to develop snapping scapula syndrome due to postoperative alteration of the biomechanics of the scapulothoracic joint [52].

A recent cadaveric study has characterized a superomedial bare area on the costal surface of the scapula between the serratus anterior insertion and the origin of the subscapularis muscle [53]. This crescent-shaped area, with an average dimension of 22.3 mm × 10.8 mm, has no overlying muscle (no subscapularis fibers). This area of the scapula may potentially lead to scapulothoracic impingement and symptomatic bursitis or crepitus.

## 6. Diagnostic Studies

**Figure 4:**
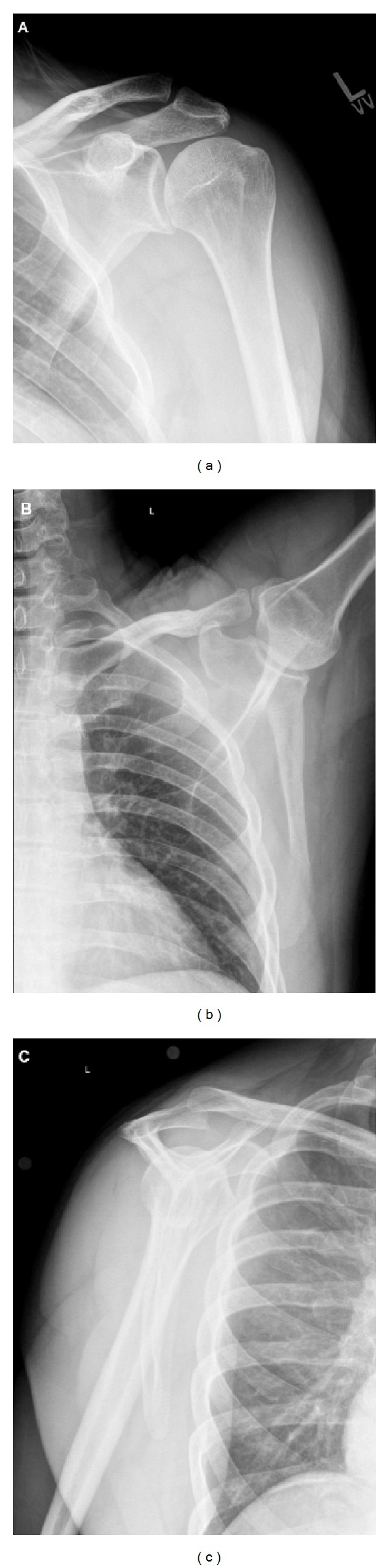
Radiographs including (a) AP, (b) axillary, and (c) scapular Y of the left shoulder.

**Figure 5:**
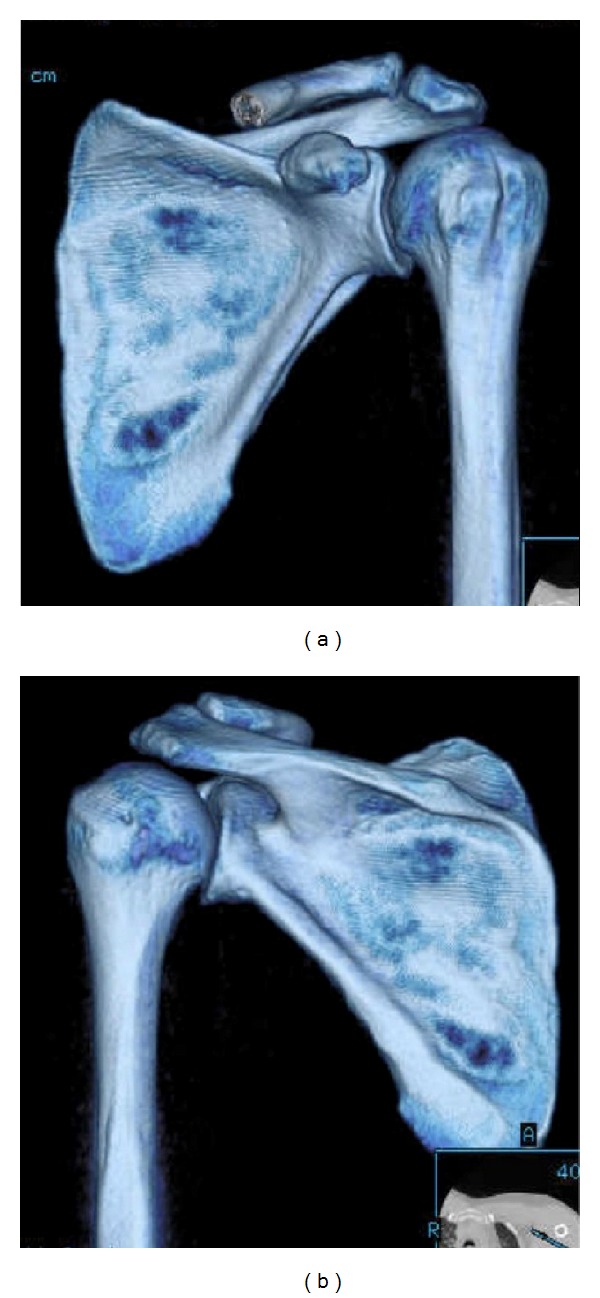
CT images with 3D reconstruction demonstrating (a) ventral surface and (b) dorsal surface of scapula.

After a thorough history and physical examination, patients presenting with complaints concerning snapping scapula syndrome often undergo a variety of imaging studies. Disorders of the scapula can be evaluated with plain radiography (Figure 4), computed tomography (CT) (Figure 5), magnetic resonance imaging (MRI), and ultrasonography [54]. Both MRI and ultrasound are more useful for evaluating bursitis, while radiographs and CT are helpful in the evaluation of bony abnormalities. Plain radiographs in the anterior-posterior, trans-scapular (scapular Y), and axillary projections can characterize the anatomical features of the scapula and the adjacent thoracic cage [39]. CT is certainly the best study for characterizing the bony morphology of the scapula. Three-dimensional CT is especially helpful in characterizing subtle bony irregularities that are often responsibly for scapulothoracic irritation and ultimately, snapping scapula syndrome [54]. MRI is the study of choice for characterizing soft tissue pathology [55, 56]. This study is especially helpful in evaluation of inflamed bursae as well as in the evaluation of potential soft tissue tumors. Though less commonly used, ultrasonography may be a cost-effective alternative, differentiating scapulothoracic bursitis from other causes of scapulothoracic pathology such as elastofibroma dorsi [34, 57].

## 7. Treatment

Nonoperative management of symptoms is the first-line of treatment for patients with scapulothoracic bursitis [2, 3, 26, 27]. Commonly used modalities include activity modification, analgesics, nonsteroidal anti-inflammatories, and physical therapy for strengthening of the periscapular musculature and rotator cuff and improvement of scapular positioning. Nonoperative management should be attempted for at least six months to one year before escalating to surgical management [58].

Patients with persistent pain and disability with impinging osseous lesions or failure of nonoperative treatment can be considered for surgical bursectomy and or superomedial angle resection. Some authors have reported that patients with symptoms at the inferomedial and superomedial borders of the scapula may be better surgical candidates [3]. Examination after injection of local anesthetic may also help to define which patients will ultimately benefit from surgery, although these bursae can be difficult to accurately inject [59–61].

**Figure 6:**
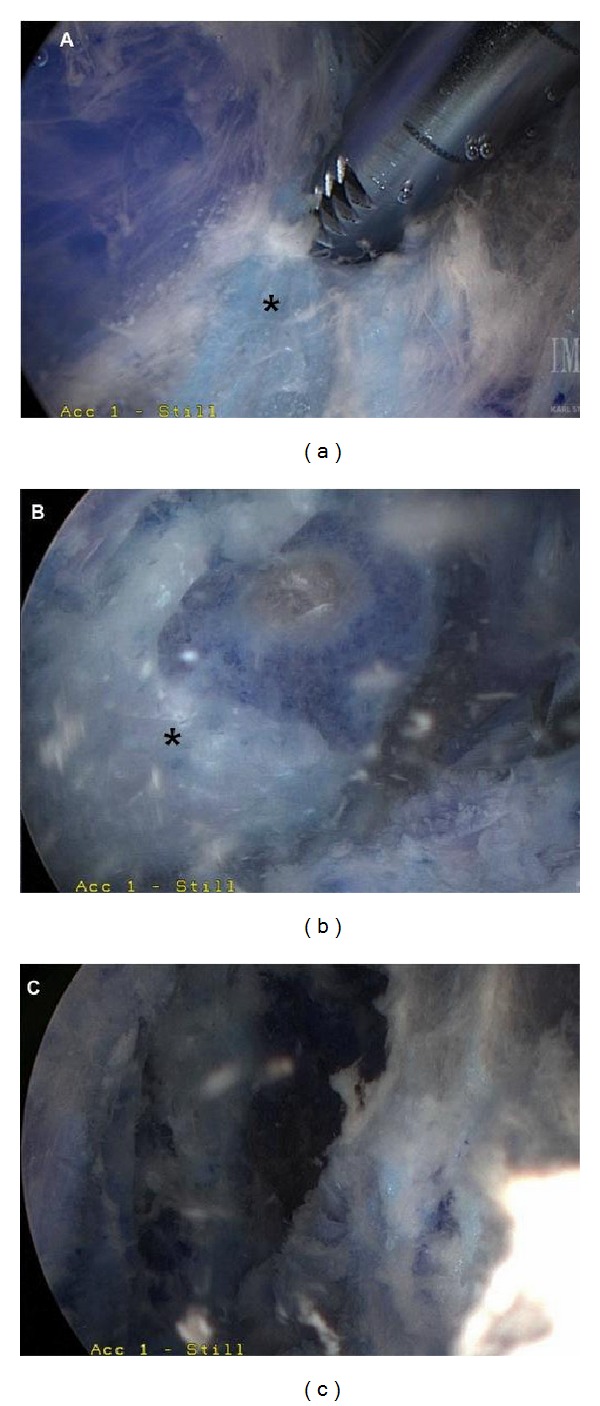
Arthroscopic images ((a), (b), and (c)) demonstrating arthroscopic bursectomy for snapping scapula syndrome with the use of an arthroscopic shaver (asterisks represent areas of inflamed bursa).

**Figure 7:**
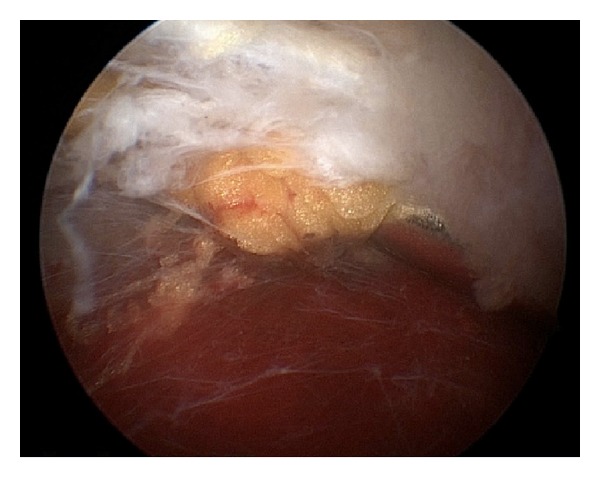
Arthroscopic image demonstrating inflamed bursa prior to arthroscopic bursectomy.

Both open and arthroscopic approaches to superomedial angle resection and bursectomy have been described [24, 29, 30, 35, 36, 47, 62, 63]. Bursae to be addressed include those adjacent to the superomedial and inferomedial angles. The patient's specific symptomology, however, will ultimately guide the location of the bursectomy. Arthroscopic-assisted and all-arthroscopic (Figures 6 and 7) technique [3, 62, 63] for snapping scapula syndrome rely on similar principles as the open procedures, and a comprehensive understanding of the anatomy described above is critical to avoid iatrogenic damage to the periscapular neurovascular structures or underlying chest wall.

## 8. Summary

The scapulothoracic articulation is a complex anatomical structure that plays a substantial role in the overall shoulder function. The osseous, ligamentous, and muscular periscapular relationships are intricate, and the underlying neurovascular anatomy can be variable. While scapulothoracic pathology is uncommon, a thorough appreciation of the anatomy, including the various muscular relationships and bursal planes, is critical for the evaluation of patients presenting with scapulothoracic disorders. Snapping scapula syndrome is caused by either osseous lesions or scapulothoracic bursitis, and appropriate recognition and treatment of these disorders are dependent on a solid foundation in periscapular anatomy.

## Figures and Tables

**Table 1:** The Seventeen Periscapular muscles.

Muscle	Origin	Insertion	Innervation
Serratus anterior	Thoracolumbar fascia, spines of vertebrae T11-T12 and L1-L2	Ribs 9–12, lateral to the angles	Long thoracic nerve
Supraspinatus	Supraspinatus fossa	Greater tubercle of the humerus (highest facet)	Suprascapular nerve
Subscapularis	Medial two-thirds of the costal surface of the scapula (subscapular fossa)	Lesser tubercle of the humerus	Upper and lower subscapular nerves
Trapezius	Medial third of the superior nuchal line, external occipital protuberance, ligamentum nuchae, spinous processes of vertebrae C7–T12	Lateral third of the clavicle, medial side of the acromion, and the upper crest of the scapular spine, tubercle of the scapular spine	Spinal accessory nerve
Teres major	Dorsal surface of the inferior angle of the scapula	Crest of the lesser tubercle of the humerus	Lower subscapular nerve
Teres minor	Upper 2/3 of the lateral border of the scapula	Greater tubercle of the humerus (lowest facet)	Axillary nerve
Triceps brachii long head	Infraglenoid tubercle of the scapula	Olecranon process of the ulna	Radial nerve
Biceps brachii	Short head: tip of the coracoid process of the scapula; long head: supraglenoid tubercle of the scapula	Tuberosity of the radius	Musculocutaneous nerve
Rhomboid major	Spines of vertebrae T2–T5	Medial border of the scapula inferior to the spine of the scapula	Dorsal scapular nerve
Rhomboid minor	Inferior end of the ligamentum nuchae, spines of vertebrae C7 and T1	Medial border of the scapula at the root of the spine of the scapula	Dorsal scapular nerve
Coracobrachialis	Coracoid process of the scapula	Medial aspect of midshaft of humerus	Musculocutaneous nerve
Omohyoid (inferior belly)	Upper border of scapula	Hyoid bone	Ansa cervicalis
Latissimus dorsi	Vertebral spines from T7 to the sacrum, posterior third of the iliac crest, lower 3 or 4 ribs, sometimes from the inferior angle of the scapula	Floor of the intertubercular groove	Thoracodorsal nerve
Deltoid	Lateral one-third of the clavicle, acromion, the lower lip of the crest of the spine of the scapula	Deltoid tuberosity of the humerus	Axillary nerve
Levator scapulae	Transverse processes of C1–C4 vertebrae	Medial border of the scapula from the superior angle to the spine	Dorsal scapular nerve
Infraspinatus	Infraspinatus fossa	Greater tubercle of the humerus (middle facet)	Suprascapular nerve
Pectoralis minor	Ribs 3–5	Coracoid process	Medial pectoral nerve
